# Thalidomide Exerts Anti-Inflammatory Effects in Cutaneous Lupus by Inhibiting the IRF4/NF-ҡB and AMPK1/mTOR Pathways

**DOI:** 10.3390/biomedicines9121857

**Published:** 2021-12-07

**Authors:** Sandra Domingo, Cristina Solé, Teresa Moliné, Berta Ferrer, Josefina Cortés-Hernández

**Affiliations:** 1 Lupus Unit, Rheumatology Departament, Hospital Universitari Vall d’Hebron, Institut de Recerca (VHIR), Universitat Autonoma de Barcelona, 08035 Barcelona, Spain; sandra.domingo@vhir.org (S.D.); fina.cortes@vhir.org (J.C.-H.); 2 Department of Pathology, Hospital Universitari Vall d’Hebron, Universitat Autònoma de Barcelona, 08035 Barcelona, Spain; teresa.moline@vhir.org (T.M.); bferrer@vhebron.net (B.F.)

**Keywords:** cutaneous lupus, thalidomide, mechanism of action, new therapy

## Abstract

Thalidomide is effective in patients with refractory cutaneous lupus erythematosus (CLE). However, the mechanism of action is not completely understood, and its use is limited by its potential, severe side-effects. Immune cell subset analysis in thalidomide’s CLE responder patients showed a reduction of circulating and tissue cytotoxic T-cells with an increase of iNKT cells and a shift towards a Th2 response. We conducted an RNA-sequencing study using CLE skin biopsies performing a Therapeutic Performance Mapping System (TMPS) analysis in order to generate a predictive model of its mechanism of action and to identify new potential therapeutic targets. Integrating RNA-seq data, public databases, and literature, TMPS analysis generated mathematical models which predicted that thalidomide acts via two CRBN-CRL4A- (CRL4^CRBN^) dependent pathways: IRF4/NF-ҡB and AMPK1/mTOR. Skin biopsies showed a significant reduction of IRF4 and mTOR in post-treatment samples by immunofluorescence. In vitro experiments confirmed the effect of thalidomide downregulating IRF4 in PBMCs and mTOR in keratinocytes, which converged in an NF-ҡB reduction that led to a resolution of the inflammatory lesion. These results emphasize the anti-inflammatory role of thalidomide in CLE treatment, providing novel molecular targets for the development of new therapies that could avoid thalidomide’s side effects while maintaining its efficacy.

## 1. Introduction

Cutaneous Lupus Erythematosus (CLE) is common and encompasses a wide range of dermatologic manifestations. As many as 70–80% of patients can develop skin lesions, which can be an early sign of systemic involvement [1,2]. CLE can be classified into specific and non-specific lesions, of which discoid lupus erythematosus (DLE) and subacute cutaneous lupus erythematosus (SCLE) are the most prevalent forms [3]. Early effective treatment may resolve the lesions, but delayed or inadequate treatment can result in permanent scarring, especially in DLE [4].

First-line therapies for CLE are antimalarial agents and/or topical steroids, together with sun protection [5]. Although most patients respond to this regimen, 30 to 40% of cases will be refractory [6]. For this minority, there is no consensus treatment algorithm, and several systemic agents have shown a variable response [7]. Thalidomide, a glutamic acid derivative with immunomodulatory and anti-inflammatory effects, has been used successfully in several oncological and chronic inflammatory dermatological conditions [8,9]. First prescribed for refractory CLE in 1975 [10], thalidomide use has increased following its reported efficacy, reaching 80–90% [6,10,11]. However, large-scale clinical trials are lacking, and serious adverse events such as teratogenicity, neurotoxicity, and thrombosis restrict its use [12,13,14,15].

Although thalidomide’s mechanism of action (MoA) has been studied, little is known of the molecular basis of its immunomodulatory effect in CLE. In vitro studies have demonstrated that thalidomide inhibits neutrophil chemotaxis, phagocytosis, angiogenesis, and production of tumour necrosis factor alpha (TNF-α). It also interacts with the T-helper response and regulation of transcription factor nuclear factor kappa-light-chain-enhancer of activated B cells (NF-ҡB) [16,17,18,19]. In the absence of an effective and safe treatment, a better understanding of thalidomide’s MoA can help to identify key target molecules for the development of new therapeutic agents.

Genome-wide gene expression profiling is increasingly used to investigate pathogenetic mechanisms and identify potential disease biomarkers [20]. RNA sequencing (RNA-seq) is a widely used method to study overall transcriptional activity. RNA-seq is a powerful investigative tool using transcriptome changes as a proxy for drug effect and has led to the discovery of potential biomarkers in several diseases, yet standard library construction is costly [21,22,23]. Using a mathematical model including the comparative RNA-sequencing data, we identified different molecular signalling signatures that provide novel insights into thalidomide’s MoA and potential therapeutic targets.

## 2. Materials and Methods

### 2.1. Patients

Six-millimetre punch skin biopsies for RNA-seq and blood samples for peripheral blood mononuclear cells (PBMCs) isolation were taken from 10 patients with active CLE, before and 4 weeks after thalidomide treatment (Table S1; see Supplementary Materials). CLE diagnosis and classification was based on clinical and histological criteria according to the 2004 Dusseldorf classification [24]. Disease activity was assessed by the validated modified CLE Disease Area and Severity Index (CLASI) [25]. The study was approved by the Vall d’Hebrón Ethics Committee and informed consent was obtained from all subjects.

### 2.2. RNA-Seq and System Biology Analysis

Whole skin RNA was extracted following the protein and RNA Isolation kit’s instructions (Thermofisher Scientific, Waltham, MA, USA). For library construction, total RNA (1 μg) was used following the Illumina TruSeq™ RNA Sample Prep Kit (Illumina, San Diego, CA, USA) manufacturer’s instructions. The resulting libraries were subjected to Illumina Hiseq 2000 sequencing platform version 3, producing 2 × 75 bp run with >65 M reads (Illumina, San Diego, CA, USA). Sequences were analyzed for quality control (FASTQC) and aligned to the Human genome (GRCh38) with STAR program V2.5.2a [26,27]. Sequencing reads were processed using the RSEM program (version 1.2.28) [28] and differential expression calculated by DESeq2 [29]. Data are available from Gene Expression Omnibus (GSE162424). We generate models to predict thalidomide’s MoA by a Therapeutic Performance Mapping System (TMPS) approach (Anaxomics Biotech, Barcelona, Spain). TPMS combines RNA-seq data with a complete characterization of CLE/Thalidomide using biological information from KEGG, Binding Database, BioGRID, REACTOME, Pubmed, Drug Bank, Stich and Supertarget [30,31,32,33,34,35,36,37] (See Supplementary Materials).

### 2.3. Flow Cytometry

PBMCs cell phenotype was analyzed by seven-colour flow cytometry (LSR Fortessa, BD Biosciences, Franklin Lakes, NJ, USA). For cell surface staining, conjugated monoclonal antibodies were used (BD Biosciences) (Table S2; see Supplementary Materials). Isotype controls were used for gate setting. Data were analyzed using FCS Express 4 Flow Research software (BD Biosciences, Erembodegem, Belgium).

### 2.4. Immunofluorescence and Immunohistochemistry

Immunohistochemistry (IHC) and immunofluorescence (IF) were performed as described on paraffin-embedded and frozen sections, respectively [37,38] using purified monoclonal antibodies listed in Table S3. Stained samples were evaluated by two blinded dermatopathologists and cell counts were quantified using Image J V1.42 (see Supplementary Materials).

### 2.5. In Vitro Ubiquitination Assay

Recombinant Human CRBN + DDB1 + CUL-4A + RBX1 (Abcam, Cambridge, UK) (1 μM), Human AMPK1 Fisher (ThermoFisher, Waltham, MA, USA) (1.5 μM) and Thalidomide (100 μM) were used with the E3 Ligase Auto-Ubiquitilylation Assay Kit (Abcam, Cambridge, UK) following the manufacturer’s instructions. Reactions were incubated at 37 °C for 2 h before separation by SDS–PAGE followed by western blot analysis.

### 2.6. Co-Immunoprecipitation for Cell-Based Ubiquitination Assay

Epidermal keratinocytes were stimulated with UVB for 6h and then treated with thalidomide. After 24 h, cells were washed twice with PBS and lysed with RIPA buffer (Sigma Aldrich, St. Louis, MI, USA) together with protease inhibitor cocktail (Sigma Aldrich, St. Louis, MI, USA). After centrifugation at 10,000 rpm for 15 min, supernatant was collected.

Concurrently, Dynabeads™ Protein G for Immunoprecipitation (Invitrogen, Waltham, MA, USA), were washed and incubated with anti-AMPK antibody (1:250) (Abcam, Cambridge, UK) in PBS 0.02% Tween™ 20 for 15 min at room temperature in order to obtain the Antibody-bead complex. Then, the mix was incubated with the obtained supernatant from the cell lysis. After 1 h at room temperature, the antibody-bead-AMPKprotein complexes were obtained. Finally, AMPK protein was eluted with elusion buffer (50 mM glycine pH 2.8) for 2 min at room temperature. Supernatants were subjected to western blot analysis for AMPK and ubiquitin protein analysis.

### 2.7. Protein Extraction and Western Blot

Skin protein samples were obtained using the PARIS kit following manufacturer’s instructions (see Supplementary Materials). Protein concentrations were determined using the BCA protein assay kit (Bio-Rad, Hercules, CA, USA). Then, 50 µg of protein was loaded into 12% SDS-PAGE and transferred to PVDF membranes (Millipore, Billerica, MA, USA) by Semi-Dry Electrophoretic Transfer (Bio-Rad, Hercules, CA, USA). Membranes were blocked with 5% BSA (RT, 1 h) followed by overnight incubation (4 °C) with specific primary antibodies (Abcam, Cambridge, UK, Table S3). Secondary HRP-labelled antibodies were added (1:500) and visualized using ECL Detection System (Santa Cruz Biotechnology, Dallas, TA, USA).

### 2.8. RNA Extraction and RT-qPCR

RNA from cultured lysed cells was obtained with RNeasy Mini Kit (Qiagen, Hilden, Germany). RNA was transcribed into cDNA with High-Capacity cDNA Reverse Transcription Kit (Applied Biosystems, Foster City, CA, USA). Gene expression was assessed by TaqMan assays (Applied Biosystems, Foster City, CA, USA) (Table S4; see Supplementary Materials).

### 2.9. Proliferation Assays

Proliferation assays were performed using CyQUANT NF Cell Proliferation Assay Kit (Invitrogen), following manufacturer’s instructions.

### 2.10. Cell Culture

Human epidermal adult Keratinocytes (HEKa) were cultured in EpiLife serum-free media with Human Keratinocyte Growth Supplement (Life Technologies, Carlsbad, CA, USA) and isolated PBMCs from healthy volunteers (Vacutainer CPT, BD Biosciences) in RPMI medium (Life Technologies, Carlsbad, CA, USA; see Supplementary Materials).

### 2.11. Gene Silencing

Third passage cultured cells at 30–50% confluence were transfected with interferon regulatory factor 4 (IRF4) or mechanistic target of rapamycin (mTOR) small interfering RNA (siRNA, ThermoFisher) or a silencer negative control (ThermoFisher, AM4615) using the lipofectamine CRISPRMAX Cas9 Reagent following the manufacturer’s instructions (ThermoFisher, Waltham, MA, USA). After 24 h, cells were treated with TNF-α (10 ng/mL) or U.V for 6 h and analyzed by qPCR-RT or immunofluorescence.

### 2.12. Co-Culture Experiments

Co-cultures were performed in modified 24-well plates with cell culture inserts (0.4-μm pore; BD Biosciences, Franklin Lakes, NJ, USA). HEKa cells were cultured at the bottom overnight. Isolated healthy donor PBMCs, stimulated with TNF-α for 6 h, were placed in the upper part of the insert and treated with thalidomide (100 ng/mL). After 24 h, the insert was removed, and HEKa cells were analyzed by immunofluorescence or RT-qPCR.

### 2.13. Statistical Analysis

Data are represented as mean ± SEM. Comparison between groups and differential gene expression was calculated with paired or unpaired t-tests as applicable using Prism GraphPad (GraphPad Software, v7.0, San Diego, CA, USA). *p* values ≤ 0.05 were considered statistically significant. RT-qPCR analysis was calculated using Fold Change differences with 2^−^^ΔΔCt^ method.

## 3. Results

### 3.1. Immunoregulatory Effects in CLE Peripheral Blood and Skin

**Figure 1:**
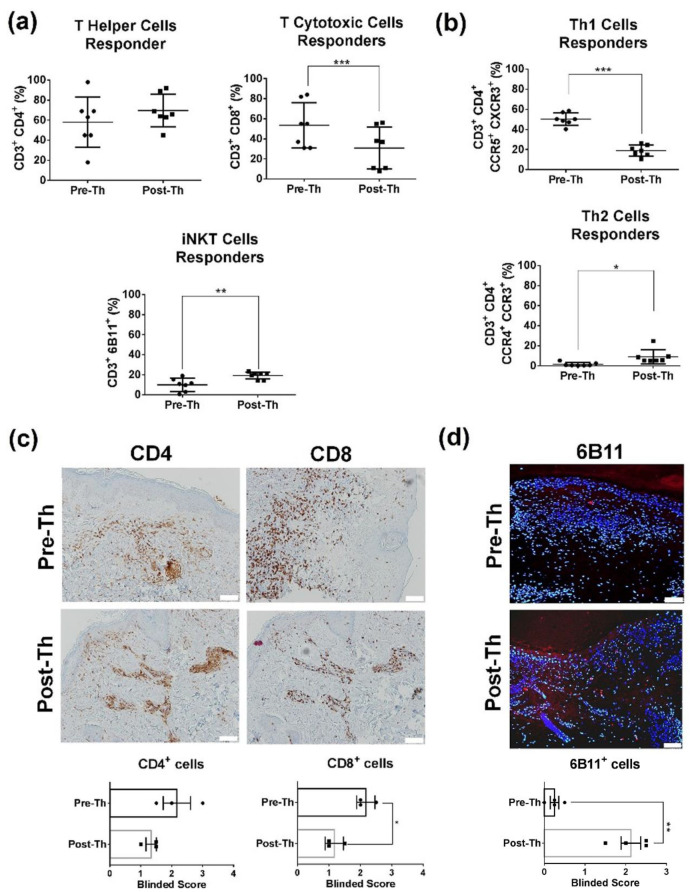
Thalidomide ameliorates skin inflammation by decreasing CD8+ T cells, increasing iNK-Tcells and promoting a Th2 response in CLE. (**a**) Flow cytometry percentages of T helper (CD3+CD4+), T cytotoxic cells (CD3+CD8+) and iNK Tcells (CD3+6B11+) in PBMCs of responder patients (*n* = 7) before and after thalidomide treatment. (**b**) Post-thalidomide, CLE patients had lower percentages of Th1 (CCR5+ CXCR3+) T cells and higher percentages of Th2 (CCR4+CCR3+). (**c**) Skin immunohistochemistry to evaluate infiltrating CD4+ and CD8+ in skin biopsies of CLE. Graphs represent the average signal intensity (*n* = 3). (**d**) Immunofluorescence of post-treatment skin samples showed a significant increase of iNK Tcells (6B11+ cells). Scale bar = 200mm. * *p* < 0.05; ** *p* < 0.005; *** *p* < 0.0001.

Ten thalidomide-treated CLE patients were included (Table S1) (see Supplementary Materials). Seven (70%) achieved clinical remission (CLASI = 0). Following treatment, responder patients had a reduction in peripheral cytotoxic CD8+ T-cells (*p* = 0.044) and an increase of iNK T-cells (*p* = 0.006) (Figure 1a). iNK T-cell related cytokines were not different after treatment; however we observed a tendency to decrease granulate cytokines (perforin A and granzyme B) in post-treatment samples (Figure S1). No significant changes in CD4+ T percentages or in dendritic cells, B-cells, or T regs were observed. Analysis of distinct Th subsets showed a skew towards a Th2 response (*p* = 0.018) (Figure 1b).

The immunohistochemical study results of the skin infiltrating cells mirrored the ones observed in peripheral blood with a significant reduction in the number of CD8+ T-cells (*p* = 0.013, Figure 1c) and an increase in iNK T-cells post-thalidomide (*p* = 0.004, Figure 1d).

### 3.2. RNA-Sequencing with Therapeutic Performance Mapping System (TMPS) Analysis Revealed Thalidomide’s Mechanisms in CLE

**Figure 2:**
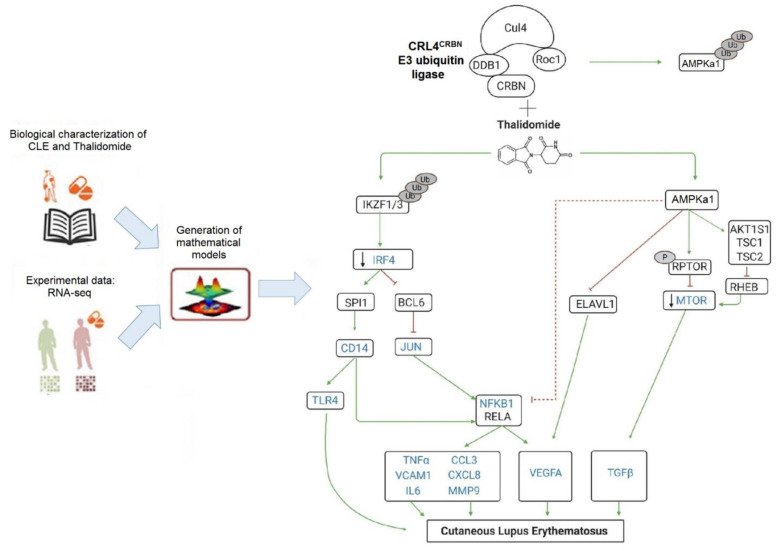
Proposed thalidomide mechanism of action in Cutaneous Lupus Erythematosus (CLE). On the one hand, in the presence of thalidomide, CRL4^CRBN^ complex ubiquitinates IKZF1/3 promoting downstream modulation of IRF4 and, on the other hand, prevents the ubiquitination of AMPKa1, increasing the expression of phosphorylated RPTOR which in turn inhibits mTOR signaling. Therefore, thalidomide modulates IRF4 and AMPK/mTOR pathways and their downstream effector molecules contributing to the resolution of inflammatory lesions in CLE.

We first identified relevant proteins in CLE pathogenesis and thalidomide through the analysis of published biological information that allowed us to establish the protein network (Tables S5 and S6; Figures S2 and S3). To obtain further insight into the molecular basis of CLE, we performed an RNA-seq. Comparative analysis of the skin RNA sequencing of thalidomide responder patients revealed 448 differentially expressed transcripts, of which 339 were protein coding genes (|log2(FC)| ≥ 1; Adj. *p* value < 0.05, data available GSE162424). To construct the Thalidomide’s MoA, the RNA-seq data was used to restrict the models (see Supplementary Materials). Finally, we identified twenty-seven differential molecules of which 14 were CLE effectors (Table S7, Figure 2). In our model, we found thalidomide to act by two CRL4^CRBN^-dependent mechanisms: (a) downregulating IRF4 leading to an inhibition of the NF-ҡB signalling pathway; and (b) regulating AMPK1/mTOR signalling pathway (Figure 2).

**Figure 3:**
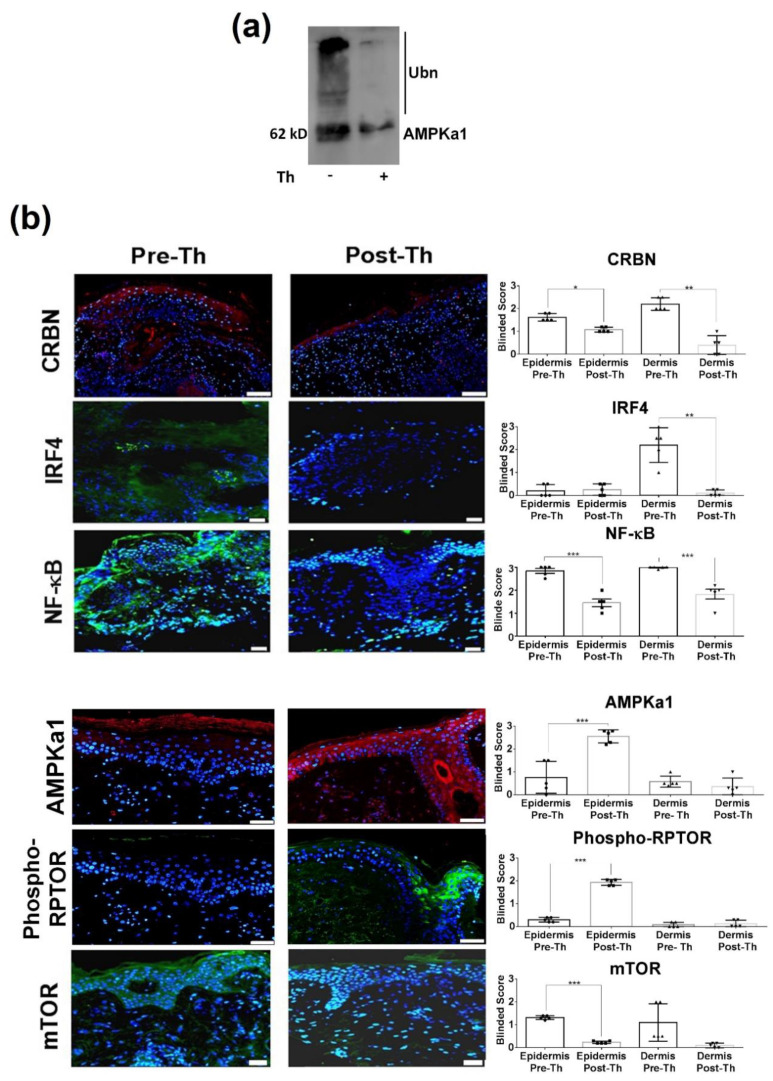
Protein levels of key target molecules identified in the analysis of the thalidomide mechanism of action. (**a**) In vitro ubiquitination of AMPKa1 by the CRL4^CRBN^ showed a reduction of the AMPKa1 ubiquitination in the presence of thalidomide. (**b**) Immunofluorescence of CLE lesional skin of paired patients showed a downregulation of CRBN (red), IRF4, NF-ҡB, mTOR (green) and upregulation of AMPK1a (red) and phosphorylated RPTOR (Phospho-Rptor, green) after thalidomide treatment. Counterstaining of nuclei is shown in blue. Average intensity fluorescence score evaluated by blinded expert pathologists in the epidermis and the dermis of the CLE skin sections (*n* = 5). Scale bar = 200mm. * *p* < 0.05; ** *p* < 0.005; *** *p* < 0.001.

In order to confirm the proposed mechanism models, we further investigated the effect of thalidomide in the CRL4^CRBN^–IKZF1/3 and AMPK1 interaction. It is well-known that in the presence of thalidomide, IKZF1/3 acts as a substrate for the CRL4^CRBN^ complex, and both Ikaros (IKZF1) and Aiolos (IKZF3) are ubiquitinated and targeted for degradation by the ubiquitin–proteasome system [38]; however, the effect of thalidomide in the CRL4^CRBN^-AMPK1 interaction is not well known. Our in vitro studies showed that in the presence of thalidomide, there was a significant reduction of the ubiquitin-dependent proteasomal degradation of AMPKa1, the catalytic subunit of the 5′-prime-AMP-activated protein kinase (AMPK) (Figure 3a).

Next, we further study the effect of thalidomide treatment in the two signalling pathways by measuring the identified key molecules at a protein level in the skin biopsies of CLE patients. Immunofluorescence in post-thalidomide skin biopsies showed a decrease expression of CRBN (*p* = 0.012 epidermis; *p* = 0.008 dermis), IRF4 (*p* = 0.0031 dermis), and NF-ҡB (*p* < 0.001 epidermis; *p* = 0.001 dermis), whereas mTOR expression was reduced primarily in the epidermal keratinocytes (*p* < 0.001, Figure 3b). Following thalidomide treatment, there was an increase of AMPKa1 (*p* < 0.001) and phosphorylated RPTOR (*p* < 0.001) protein expression levels in the epidermis (Figure 3b). Results were confirmed also by western blot (Figure S4).

### 3.3. Thalidomide Modulates PBMCs via the IRF4/NF-ҡB Signalling Pathway

**Figure 4:**
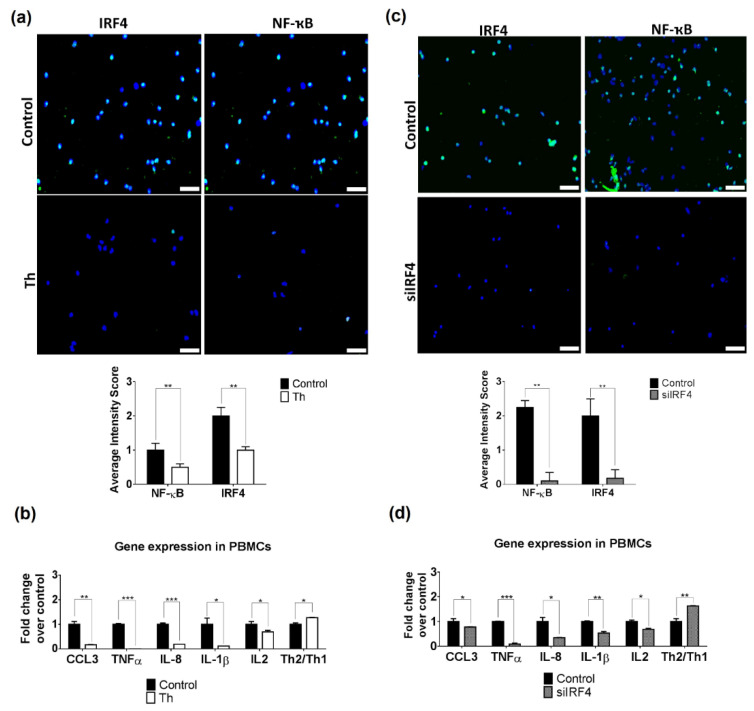
Thalidomide modulates the IRF4/NF-ҡB pathway in PBMCs. (**a**) Immunofluorescence of NF-ҡB and IRF4 protein levels (green staining) in PBMCs treated with thalidomide (Th) or with PBS + 1%DMSO (control conditions). Dapi was used to stain nuclei of cells (blue). (**b**) RT-qPCR of NF-ҡB inflammatory effectors CXCL3, TNFɑ, IL-8, IL-1β and IL2 was performed in PBMCs treated with or without thalidomide. Ratio T helper 2 vs. 1 was evaluated via gene expression of their transcription factors. (**c**) IRF4-silenced PBMCs were stained in order to evaluate NF-ҡB and IRF4 protein levels (green). Control condition was performed using a non-targeting siRNA. (**d**) Gene expression in IRF4-silenced PBMCs were determined by RT-qPCR. Fold change was calculated over control conditions. GADPH was used as endogenous control. Scale bar = 50 µm. * *p* < 0.05, ** *p* < 0.005, *** *p* < 0.001.

Our immunofluorescence findings indicate that thalidomide may modulate the IRF4 pathway in the dermal inflammatory infiltrates. We performed in vitro experiments with stimulated PBMCs treated with thalidomide that showed a significant reduction of IRF4 (*p* < 0.01) and NF-ҡB (*p* < 0.01) expression but no changes were observed in mTOR (Figure 4a and Figure S5). Gene expression analysis of final effector molecules showed a significant reduction of NF-ҡB-related cytokines (*IL-1β*, *IL-8* and *TNFα* (9.09, 5.25 and 33.3-fold decrease, respectively) and *CCL3* (6.25-fold decrease). The analysis of the T helper subsets showed an increase of the Th2/Th1 ratio (*GATA3/T-bet*) (1.26 and 1.63-fold increase, respectively) with a significant reduction of *IL-2* levels (1.45-fold decrease, Figure 4b). No significant changes were found in PBMCs proliferation and autophagy (Figures S6 and S7).

To demonstrate that the thalidomide anti-inflammatory effect in PBMCs is dependent on IRF4 modulation, we silenced IRF4. We showed that IRF4 silencing induced similar results to the ones observed in thalidomide-treated cells with a reduction of NF-ҡB protein levels (*p* < 0.01) and a downregulation of *IL-1β*, *IL-8*, *TNFɑ* (1.87, 2.89 and 11.04-fold decrease, respectively) and *CCL3* expression levels (1.27-fold decrease, Figure 4b). siIRF4 PBMCs showed also a shift of the Th1/Th2 balance with an increase of the Th2/Th1 ratio and a significant reduction of *IL-2* levels.

### 3.4. Thalidomide Modulates the AMPK1/mTOR-NF-ҡB Signalling Pathway in Keratinocytes

**Figure 5:**
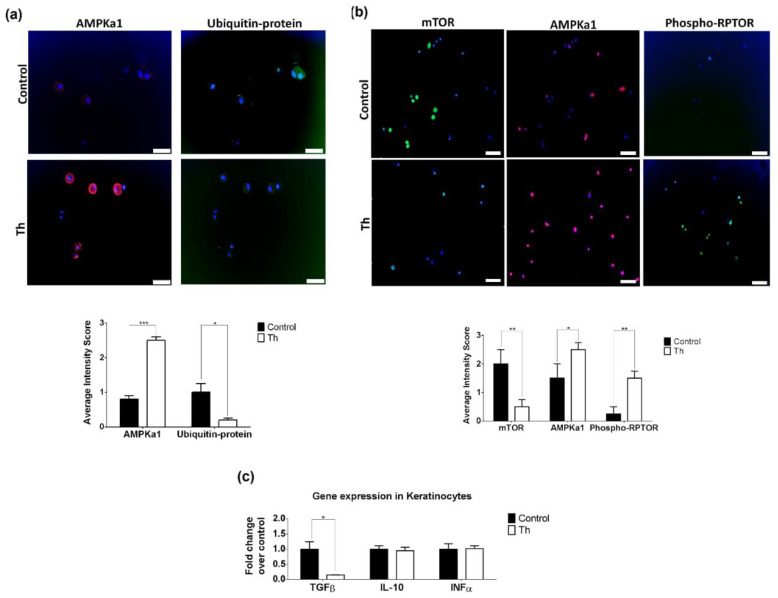
Thalidomide modulates the AMPKa1/mTOR in keratinocytes. (**a**) In vivo ubiquitination was performed in keratinocytes treated or non-treated with thalidomide. Immunofluorescence of AMPKa1 (red) or ubiquitin-proteins conjugates (green) revealed that in the presence of thalidomide AMPKa1 was not degraded. Scale bar = 50 µm. * *p* < 0.05, *** *p* < 0.0005. (**b**) Protein levels of AMPKa1 (red), mTOR and phosphorylated RPTOR (Phospho-RPTOR, green) were measured using immunofluorescence in keratinocytes treated with thalidomide (Th) or with PBS+1%DMSO (control conditions). Nuclei of cells were marked with dapi (blue stainning). Scale bar = 50 µm. * *p* < 0.05, ** *p* < 0.005. (**c**) RT-qPCR of mTOR inflammatory effectors TGFβ, IL-10, INFα was performed in UVB-treated keratinocytes in the presence or not of thalidomide. Fold changes were calculated over control. * *p* < 0.05.

mTOR epidermal expression in pre-treated samples led us to study thalidomide’s effect on keratinocytes through this signalling pathway. First, we demonstrated at a tissue level the effect of thalidomide in AMPK. Keratinocyte cell-based ubiquitination was performed in the presence or absence of thalidomide. A significant increase of AMPKa1 protein levels were observed in thalidomide-treated keratinocytes in comparison to control conditions (*p* < 0.001, Figure 5a). Simultaneously, a significant reduction of ubiquitin-protein conjugates were observed suggesting that ubiquitination of AMPKa1 is more pronounced in the absence of thalidomide (*p* = 0.0201, Figure 5a). This observation was also confirmed by western blot (Figure S8). mTOR expression levels decreased in UVB-stimulated keratinocytes following thalidomide (*p* = 0.009, Figure 5b). Conversely, upregulation of AMPKa1 and phosphorylated RPTOR expression levels were observed (*p* = 0.036 and *p* = 0.003, respectively, Figure 5b). The gene expression analysis of downstream mTOR-dependent cytokines (IL-10, TGFβ and INFα) in thalidomide-treated keratinocytes only showed a significant reduction of TGFβ (6.69-fold decrease, Figure 5c). Keratinocyte proliferation, apoptosis and autophagy after thalidomide were analysed and no changes were observed (Figures S9 and S10).

**Figure 6:**
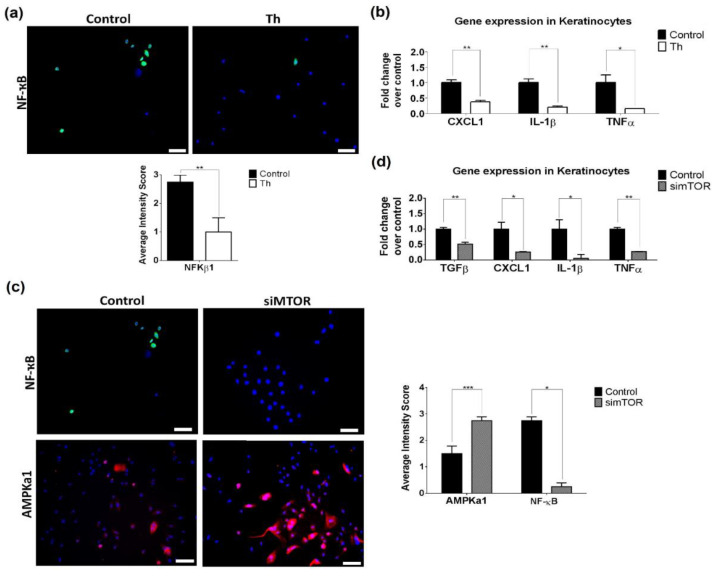
Thalidomide effect in keratinocytes is dependent on mTOR. (**a**) Immunofluorescence showed a reduction of NF-ҡB protein levels in keratinocytes in the presence of thalidomide (green staining). Scale bar = 50 µm. ** *p* < 0.005. (**b**) A reduction of NF-ҡB-related cytokine gene expression was confirmed by RT-qPCR analysis. Fold change was calculated over control conditions. GADPH was used as endogenous control. * *p* < 0.05, ** *p* < 0.005. All the experiments were performed in triplicate (**c**) AMPK1a and NF-ҡB protein levels were measured by immunofluorescence in silenced mTOR keratinocytes and control keratinocytes (non-specific silenced gene). Scale bar = 50 µm. * *p* < 0.05, *** *p* < 0.0005. (**d**) Gene expression of related cytokines were measured by RT-qPCR analysis in silenced mTOR keratinocytes (simTOR). Fold change was calculated over non-silenced mTOR keratinocytes (control conditions). * *p* < 0.05, ** *p* < 0.005.

We also studied the ability of thalidomide to modulate NF-ҡB in keratinocytes, since epidermal NF-ҡB levels were significantly reduced in skin biopsies following thalidomide treatment. The treatment of these cells with thalidomide reduced significantly the NF-ҡB protein levels (*p* = 0.005, Figure 6a). Furthermore, gene expression analysis of NF-ҡB-dependent cytokines (*TNFα*, *IL8*, *IL1β*, *IL6*, *CXCL1* and *MMP9*) showed a reduction of *IL-1β* (5-fold decrease), *TNFα* (6.71-fold decrease) and *CXCL1* (2.67-fold decrease) (Figure 6b). This NF-ҡB reduction was also observed in keratinocytes when mTOR was silenced, along with an increase of AMPKa1 protein levels (*p* < 0.05) (Figure 6c). Downregulation of *TGFβ* (1.92-fold decrease), *IL-1β* (19.88-fold decrease), TNFα (3.70-fold decrease) and *CXCL1* (4.29-fold decrease) gene expression levels were also observed (Figure 6d).

Silencing IRF4 in keratinocytes had no effect in NF-ҡB protein levels (Figure S11), reinforcing the evidence of a crosstalk between NF-ҡB- and AMPK/mTOR-signaling pathway.

### 3.5. Thalidomide-Treated PBMCs Downregulate Keratinocyte mTOR Signalling Pathway

**Figure 7:**
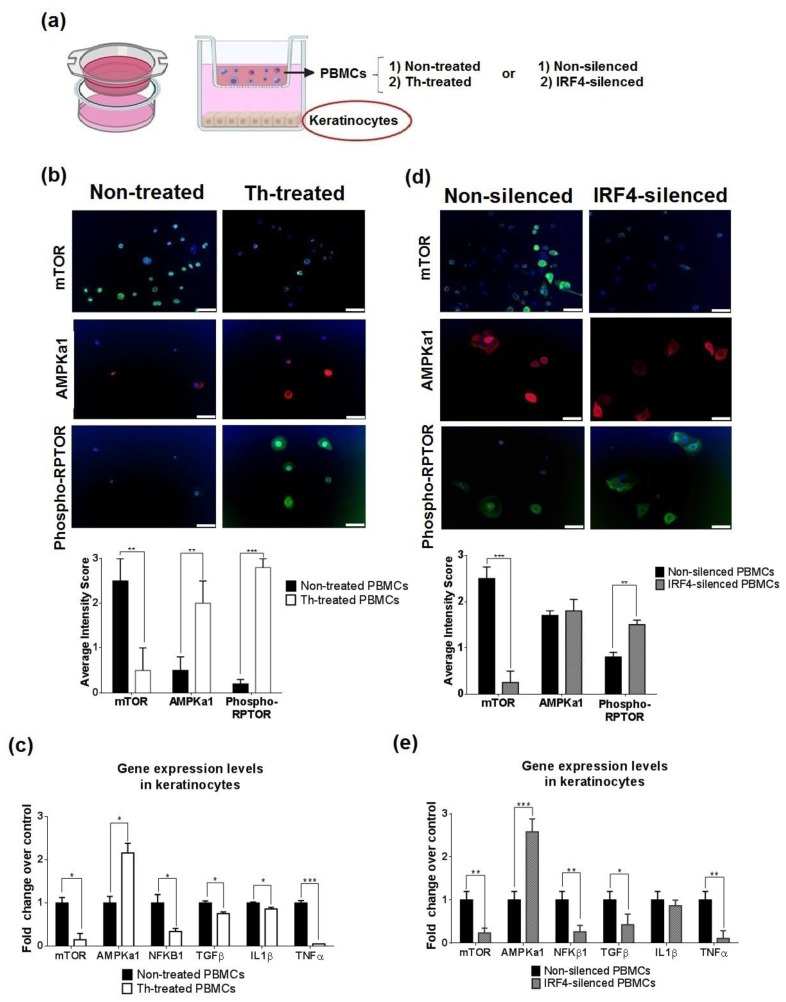
In vitro co-culture of PBMCs and keratinocytes revealed a predominance of IRF4-thalidomide effect. (**a**) PBMCs were stimulated with TNFα and treated or non-treated with thalidomide (Th) and co-cultured with keratinocytes. After 24 h of co-culture, keratinocytes were analysed by immunofluorescence and gene expression assays. A similar experiment was performed using non-silenced or IRF4-silenced PBMCs (siIRF4). (**b**) mTOR protein levels (green) were significantly decreased in the presence of Th-treated PBMCs, whereas AMPKa1 (red) and phosphorylated RPTOR (green) were increased after thalidomide treatment. (**c**) Keratinocyte gene expression levels of the mTOR-related molecules and inflammatory effectors were measured by RT-qPCR. (**d**) mTOR protein levels in keratinocytes also decreased in the presence of IRF4-silenced PBMCs. Phosphorylated-RPTOR increased in keratinocytes after PBMCs were IRF4 silenced (**e**) mTOR related molecules and inflammatory effectors were measured by RT-qPCR in keratinocytes after co-cultured with non-silenced or IRF4-silenced PBMCs. Scale bar = 50 µm. * *p* < 0.05, ** *p* < 0.005, *** *p* < 0.001.

As the interaction between epithelial cells and the immune system is tightly regulated, we performed cross-talking in vitro functional studies between thalidomide-treated PBMCs and keratinocytes (Figure 7a). Thalidomide-treated PBMCs co-cultured with keratinocytes produced a significant downregulation of keratinocyte mTOR protein levels and an increase of the inhibitor AMPKa1 and phosphorylated mTOR (Figure 7b). Analysis of gene expression levels showed the reduction of MTOR (6.6-fold decrease), an increase of AMPKa1 (2.20-fold increase), a decrease of NFKB1 (2.94-fold decrease) and related cytokines (TGFβ, IL1β and TNFα) (Figure 7c). Cross-talking studies using IRF4-silenced PBMCs also showed the same effect in mTOR and phosphorylated mTOR protein levels (Figure 7d). Gene expression levels of MTOR, NFKB1, TGFβ, TNFα (4.24, 3.84, 2.36 and 9.13-fold decrease, respectively) and AMPKa1 (2.58-fold increase, Figure 7e).

## 4. Discussion

Our study examined thalidomide’s immunomodulatory mechanism in cutaneous lupus. CLE has a distinctive T-cell signature with an imbalance towards a Th1 response [39] and CD8+ T-cell predominance in early inflammatory stages [40]. Thalidomide induced a reduction of cytotoxic CD8+ T-cells and increased the number of iNKT cells both circulating and in tissue. Activated cytotoxic lymphocytes (CTLs), like cytotoxic CD8+ T-cells, contribute to basal keratinocyte damage and inflammatory infiltration in CLE, especially at the dermo-epidermal junction, and correlate with IFN-α expression and damage extension [41]. iNKT cells are a subset of unconventional T-cells which recognise the MHC class I-like CD1d protein with the expression of an invariant TCR chain (Vα24-Jα18) paired with a Vβ11 chain [42]. Lupus patients, especially those with severe cutaneous involvement, have a numerical and functional reduction of circulating iNKT cells, but enrichment has been described in lesional skin. In line with previous IMiDs studies [43], during lesion resolution, we found an increment in both tissue and circulating iNKT cells after thalidomide. The exact role of these cells is not completely understood since they are functionally versatile and may mediate both pathogenic and regulatory immune functions. Whereas, on the one hand, iNKT cells participate in the pathogenesis of several skin inflammatory disorders producing interferon gamma and IL-4 [44], we did not find a difference in their related cytokines. On the other hand, iNKT has been described as potent downregulators of CD8+ cytotoxic T cells [45]; they are implicated in skin would healing [46,47] and they alleviate lupus dermatitis in an MRL-lpr/lpr model [48]. Modulation of the different Th subsets by IMiDs has also generated opposing data [49,50,51]. In our study, thalidomide induced a Th2 response both in vivo and in vitro. Some Th2 responses are related to the expression of wound healing genes and growth factors involved in tissue regeneration [52], so Th2 enhancement may contribute to skin repair.

To further investigate the thalidomide MoA, we combined machine learning approaches with CLE RNA-sequencing data to obtain a novel predictive model. We showed that thalidomide modulates CLE by targeting two CRL4^CRBN^-dependent pathways, downregulating IRF4 via IKZF1/3 and mTOR through regulation of AMPK1 activity. The study confirmed previous reports describing CRBN as the primary target of thalidomide [15,53] and its expression decreased following treatment both in the dermal inflammatory infiltrates and epidermis. CRBN functions as a substrate receptor for the cullin-4-containing E3 ubiquitin ligase complex CUL4–RBX1–DDB1 (CRL4A) and is responsible for the recruitment of substrates for degradation by the ubiquitin-proteasome pathway. IMiDs bind to CRBN and alter the substrate specificity of CRL4^CRBN^ blocking the degradation of proteins involved in angiogenesis, tumoral activity and inflammation [54,55] but also inducing teratogenicity [56].

As in other inflammatory skin conditions like psoriasis vulgaris, acne, atopic dermatitis, and hidradenitis suppurative [57,58], we found in active lesions of CLE an increased expression of IRF4 in the dermis and mTOR in the epidermis. Following thalidomide there was a reduction in these expression levels. Further in vitro experiments confirmed the effect of thalidomide through the two different signalling pathways according to the skin cell type. Thalidomide reduced IRF4 signalling in lymphocytes whereas the effect on mTOR was observed in keratinocytes.

IRF4 is a member of the IRF family of transcription factors, expressed in immune cells relevant in the IFN signature [59]. IRF4 is required for proper maturation and differentiation of immune cells [60]. IRF4 dysregulation has been described in rheumatoid arthritis and SLE and it is associated with initiation and disease progression [61]. IMiDs can induce CRL4^CRBN^-dependent degradation of the Ikaros family zinc finger protein-1 (IKZF1, Ikaros) and 3 (IKZF3, Aiolos), two transcription factors involved in lymphoid development and differentiation and highly expressed in B cell malignancies, leading to an inhibition of IRF4 expression at transcriptional level [38,62,63]. We showed that thalidomide modulates the IRF4/NF-ҡB signalling pathway in PMBCs and contributes to the resolution of inflammation by reducing the expression of NF-ҡB and its dependent cytokines and chemokines IL-1β, IL-8, TNFα and CCL3.

**Figure 8:**
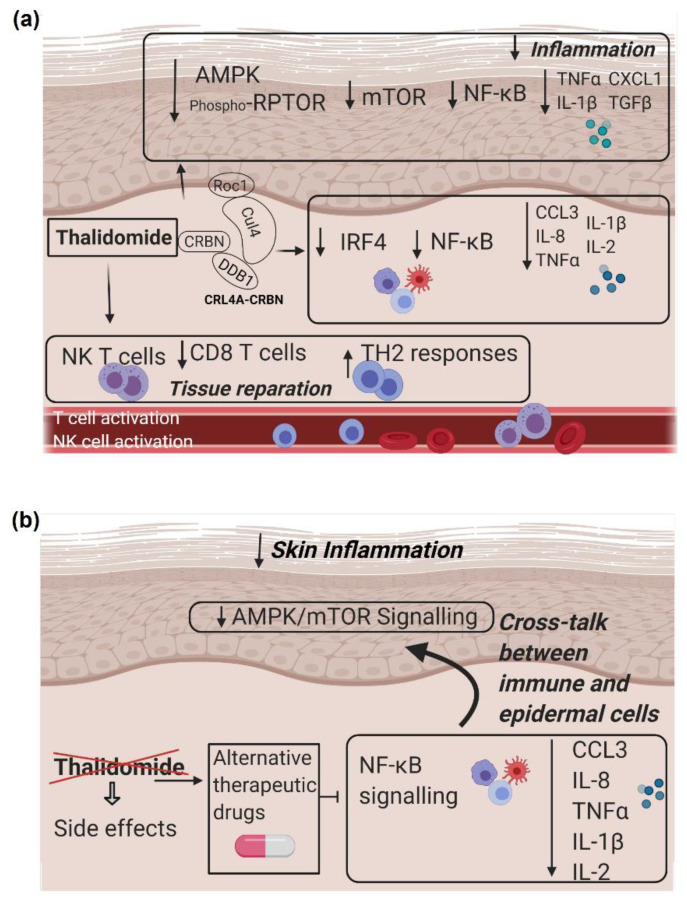
Thalidomide as alternative therapy in CLE. (**a**) Thalidomide binds CRBN in the cullin-4 E3 ubiquitin ligase complex (CRL4^CRBN^) and promotes: A downregulation of mTOR protein, by reducing the AMPK ubiquitination and increasing the RPTOR phosphorylation that downregulated NF-ҡB and its related cytokines in keratinocytes. Also promoted is a reduction of IRF4 expression in lymphocytes that decreases the expression of NF-ҡB and related cytokines. In addition, thalidomide enhances tissue reparation promoting Th2 responses, iNK T cells and lower prevalence of CD8+ T cells. (**b**) Alternative therapeutic drugs targeting NF-ҡB signalling may avoid its important side effects and maintain its anti-inflammatory properties.

AMP-activated protein kinase (AMPK) has also been identified as a CRBN-binding protein [64]. AMPK is an important intracellular energy sensor and is activated by phosphorylation of threonine at position 172 (Thr 172) of the α subunit. CRL4^CRBN^ down-regulates the total quantity of the AMPK α subunit by polyubiquitination. Previous reports have shown that thalidomide markedly stimulates the activation of AMPK [63,64] and reduces AMPK α polyubiquitination [65,66,67]. Accordingly, we demonstrated that thalidomide reduced the AMPK α ubiquitination in a CRBN-dependent manner and increased its expression. Consequently, we observed an increase of RPTOR phosphorylation and a reduction of mTOR signalling. mTOR is a serine threonine kinase crucial in skin homeostasis and morphogenesis, especially in the regulation of keratinocyte differentiation and epidermal stratification [68]. There are two biochemically distinct mTOR complexes, mTORC1 and mTORC2. The activity of mTORC1 is suppressed by AMPK by directly phosphorylating at least two regulator proteins, tuberous sclerosis 2 (TSC2) and RPTOR. In vitro studies showed that treatment with thalidomide or simTOR significantly reduced keratinocyte-derived cytokines TGFβ, IL-1β, TNFα and CXCL1 (Figure 8a) contributing to the resolution of inflammation. In addition, we showed that the specific inhibition of mTOR decreased NF-ҡB expression in keratinocytes. The existence of a crosstalk between mTOR and NF-κB has been described in other cellular types [69]. Not only have we described a crosstalk between mTOR and NF-κB in keratinocytes, but we have also shown the ability of thalidomide-treated PBMCs to reduce the expression of mTOR and related cytokines in co-culture studies.

Our previous work in DLE [70] and this study confirm the relevance of NF-ҡB in CLE. NF-ҡB was the common target molecule in which thalidomide acted through different signalling pathways in their respective skin cells [71]. NF-kB is a key player in the control of both innate and adaptive immunity. NF-kB activity is essential for lymphocyte survival, activation, and mounting normal immune responses. Constitutive activation of the NF-kB pathway is often associated with inflammatory diseases like rheumatoid arthritis, inflammatory bowel disease, multiple sclerosis, and asthma [72]. Activation of NF-ҡB in keratinocytes has been reported in psoriasis lesions resulting in the production of multiple inflammatory molecules that initiate and sustain the inflammatory process [73,74,75,76]. In addition, it has been demonstrated that topical application of an NF-ҡB inhibitor improved atopic dermatitis in NC/NgaTnd mice [77]. Together, these data support the further study of NF-ҡB as novel a therapeutic target [78]. While global inhibition may result in profound side effects by selectively targeting specific NF-ҡB subunits or signalling components relevant to a particular disease, toxicity can be minimized.

## 5. Conclusions

Taken together, we demonstrated that thalidomide’s immunomodulatory anti-inflammatory effect in CLE comprises several mechanisms that include a reduction of predominantly CD8+T cells, and a switch from Th1 to Th2 response. Furthermore, thalidomide reduced NF-ҡB related inflammatory cytokines and chemokines via the modulation of IRF4- and AMPK/mTOR-signalling pathways. Targeting the function of these key molecules may be an alternative to thalidomide for the treatment of CLE (Figure 8b).

## Data Availability

Data are available from Gene Expression Omnibus (GSE162424) at https://www.ncbi.nlm.nih.gov/geo/query/acc.cgi?acc=GSE162424 (accessed on 20 October 2021).

## Supplementary Materials

The following are available online at www.mdpi.com/article/10.3390/biomedicines9121857/s1, Figure S1: PBMCs from patients were extracted pre and post-thalidomide (*n* = 5), Figure S2: Topological analysis represents the relationships between proteins in the mechanism of action over cereblon modulation, Figure S3: Topological analysis represents the relationships between proteins in the mechanims of action over IRF4, Figure S4: Western blot of lysates from paired skin biopsies, Figure S5: Immunofluorescence of mTOR in thalidomide-treated or non-treated PBMCs, Figure S6: Proliferation in healthy PBMCs after thalidomide treatement, Figure S7: Immunofluorescence in thalidomide-treated or non-treated PBMCs to monitoring autophagy, Figure S8: Western blot of cell based ubiquitination assay in keratinocytes terated or non-treated with thalidomide, Figure S9: Profiferation and apoptosis of human epidermal keratinocytes after thalidomide addition, Figure S10: Immunofluorescence in thalidomide-treated or non-treated keratinocytes to monitoring autophagy, Figure S11: Immunofluorescence of NF-ҡB in cultured keratinocytes with thalidomide or silenced IRF4, Table S1: Clinical and laboratory characteristics of the study subjects, Table S2: Conjugated antibodies used in flow cytometry analysis, Table S3: Antibodies used in immunofluorescence, immunohistochemistry and western blot analysis, Table S4: Primer IDs used in Taqman RT-qPCR from Applied Biosystems, Table S5: Characterization of cutaneous lupus erythematosus (CLE) includes 206 proteins distributed in the named motives, Table S6: Detail of the targets identified for thalidomide and the use of the targets in the different project steps, Table S7: MoA included 27 proteins with 14 proteins related directly with CLE.
