# Supplementary material for: Thalidomide Exerts Anti-Inflammatory Effects in Cutaneous Lupus by Inhibiting the IRF4/NF-ҡB and AMPK1/mTOR Pathways
Source: Biomedicines. 2021 Dec 7;9(12):1857. doi: 10.3390/biomedicines9121857 (PMC8698478; doi:10.3390/biomedicines9121857)
Supplement: Supplementary file 1 [file biomedicines-09-01857-s001.zip › biomedicines-1452778-supplementary.pdf]

Supporting Information

Thalidomide exerts anti-inflammatory effects in cutaneous lupus via inhibiting the IRF4/NF-κB and AMPK1/mTOR pathway

Sandra Domingo[1], Cristina Solé[1], Teresa Moliné[2], Berta Ferrer[2], Josefina Cortés-Hernández[1]

[1]Lupus Unit, Rheumatology Department, Hospital Universitari Vall d´Hebron, Institut de Recerca (VHIR), Universitat Autònoma de Barcelona, Barcelona, Spain.

[2]Department of Pathology, Hospital Universitari Vall d´Hebron, Universitat Autònoma de Barcelona, Barcelona, Spain

1. SI Materials and Methods

Patients' clinical characteristics and samples

A total of 10 patients were included in the study. Demographic characteristics are shown in Table S1. At the time of skin biopsy, disease activity and degree of scarring was assessed by the validated modified CLE Disease Area and Severity Index (CLASI) (Albrecht et al., 2005) Patient's inclusion criteria included: age ≥ 18 years old, the presence of skin lesional area bigger than 3cm, a validated CLE Disease Area and Severity Index (CLASI) greater than 4 and no previous treatment with immunosuppressants for ≥1 month or topical corticoids for at least ≥2 weeks. The study was approved by the Local Vall d'Hebrón Ethics Committee and informed consent was obtained from all subjects before the study.

At inclusion all patients received oral thalidomide (100 mg/day) at night for 4 weeks. A six-millimetre punch biopsy was taken from lesional skin from CLE untreated patients and another six-millimetre punch biopsy was taken from paired patient's post-thalidomide treatment. The skin punch was divided into three sections: the first section was used for RNA-sequencing experiments, the second was immediately frozen in liquid nitrogen in OCT compound for immunofluorescence studies and the third was fixed in 5% formalin and paraffin-embedded in order to perform immunohistochemistry techniques.

RNA library construction and sequencing

Total RNA from skin biopsies was obtained using RNeasy Mini Kit (Qiagen, Hilden, Germany) and ribosomal RNA was removed using Epicentre's Ribo-Zero rRNA Removal kit (Illumina, San Diego, USA). RNA integrity was evaluated using Bioanalyzer 2100 obtaining values ≥8.5 (Agilent Technologies, Santa Clara, CA, USA). Samples were converted to cDNA and subsequently subjected to fragmentation, linker adapter ligation and amplification using TruSeq library generation kits (Illumina, San Diego, USA) according to manufacturer's instructions. The constructed libraries were amplified using 8 cycles of PCR. The resulting libraries were subjected to Illumina Hiseq 2000 sequencing platform version 3 producing 2x75 bp run with >65 M reads (Illumina, San Diego, USA).

Image analysis, sequencing quality evaluation, and data production summarisation were performed using the Illumina/Solexa pipeline (Illumina, San Diego, USA). Sequences were analyzed for quality control (FASTQC) and aligned to the Human genome (GRCh38) using STAR program (version 2.5.2a) (Wingett et al., 2018; Dobin et al., 2013). RSEM program (version 1.2.28) (Li et al., 2011) was used to determine transcript assembly, and the abundance and expression levels were determined based on the fragments per kb per million (FPKM) values, a way of normalizing read counts by calculating the number of reads mapped to each transcript divided by its length and the total number of mapped reads in the sample. To find differentially expressed genes and transcripts, the logarithmic ratios of FPKMs were calculated by pairwise comparisons of the expression between pre- and post-skin thalidomide samples with tests for significant differences using DESeq2 (Love et al., 2014). To obtain high-quality DEGs, we set the threshold for the false-discovery rate at < 0.05 and for fold change at ≥ 2 or ≤ 05 (|log2FC| ≥ 1) in the comparison analysis.

TPMS technology [31]

The Therapeutic Performance Mapping System (TPMS) is a tool that creates mathematical models of a drug/pathology protein pathways to explain a clinical outcome or phenotype (Anaxomics Biotech, Barcelona, Spain). These mathematical models find mechanism of action (MoAs) that explain how a *Stimulus* (i.e. proteins activated or inhibited by a drug) produces a *Response* (i.e. proteins active or inhibited in a phenotype). The detailed steps are explained below:

1. Molecular characterization of CLE disease and thalidomide

To apply the TPMS approach and create the mathematical models of MoAs, a characterization of CLE disease and thalidomide is needed. We manually curated a list of proteins and motives relevant for cutaneous lupus erythematosus (CLE) pathogenesis and targets for thalidomide's mechanism of action (Supplemental Table 5 and 6). Manual curation was performed through an extensive and careful review of full-length articles in the PubMed database, Drug Bank, Stitch and Supertarget (Knehisa et al., 2014; Gilson et al., 2016; Chatr-Aryamontri et al., 2017; Croft et al., 2014). The search was expanded using the "related articles" function and article reference list. For CLE characterization, we included 206 proteins. For thalidomide molecular drug characterization, eight main molecules have been identified (CRBN, IKZF1, IKZF3, IRF4, MEIS2, ORM1, ORM2, FGF2) and 36 proteins were related to them.

2. Generation of mathematical models

We generated a biological map between CLE proteins and thalidomide targets using public information about protein-to-protein interactions, physical interactions and modulations, signaling, metabolic relationships and gene expression regulation that are founded in: KEGG, Binding Database, BioGRID and REACTOME (Jorba et al., 2020; Wishart et al., 2008; Szklarzyk et al., 2016; Hecker et al., 2012).

The algorithm of TPMS for generating the mathematical models is similar to a Multilayer Perceptron of an Artificial Neural Network over the biological map (where neurons are the proteins, and the edges of the network are used to transfer the information). It takes as input signals the activation (+1) and inactivation (-1) of the drug target proteins and as output proteins implicated in CLE pathogenesis.

The models have to be able to weigh the relative value of each protein (node) relation. Since the number of links is very high, the number of parameters to solve also increases exponentially. Anaxomics applied Artificial Intelligence (AI) technologies for modelling complex network behaviors, including graph theory and statistical pattern recognition technologies; genetic algorithms; artificial neural networks; dimensionality reduction techniques; and stochastic methods like Simulated Annealing, Monte Carlo among others (Anaxomics Biotech, Barcelona, Spain).

3. Molecular mechanism construction

Then a collection of restrictions, defined as the true set of edges and nodes with the property of being active or inactive, are used for validating the mathematical models obtained with TPMS (Truth table). Two type of restrictions are used: 1) information found in microarray database (GEO, PHOSIDA, 2D gel database, BED) and drug database (DrugBank); 2) data obtained from our RNA-seq analysis using skin biopsies of CLE patient's pre and post-thalidomide treatment

As the number of restrictions is always smaller than the number of parameters required by the algorithm, any process modelled by TPMS has a "population" of different solutions, which is set around 10^6–10^9, since this interval is estimated to faithfully portray nature. From this set of solutions, only the best ones (showing acceptable accuracy values for the Truth Table) are used to construct a "global" or "average" molecular mechanism, which represents the most probable molecular mechanism according to the current biological knowledge. In the present work, two MoAs were detected as the best: molecular response to thalidomide downstream 1) its main target (Cereblon) and 2) indirect modulation of IRF4 activity. The graphical representation of the interactions between the proteins in the two best MoAs are showed in Figure S3 and S4, respectively.

Evaluation of immunohistochemistry and immunofluorescence skin sections

Immunohistochemistry and immunofluorescence results were evaluated on blinded specimens by two independent dermatopathologists from the Vall d'Hebron pathology unit. Positive cells per millimetre were quantified using computer-assisted image

analysis software (ImageJ 1.42, National Institutes of Health, Bethesda, MD, USA). The staining of the epidermis, dermis and inflammatory infiltrate was evaluated semi-quantitatively using the following blinded score: 0 (<10% positive cells), 0.5 (10-20% positive cells), 1 (20-40% positive cells), 1.5 (40-60% positive cells), 2 (60-80% positive cells), 2.5 (80-90% positive cells) or 3 (>90% positive cells).

PBMCs isolation

Blood from CLE patients before and after thalidomide treatment and healthy controls was collected directly into mononuclear cell preparation tubes with sodium citrate (Vacutainer CPT, BD Biosciences). Tubes were centrifuged at 3000 rpm for 30 minutes at room temperature (RT). After that, the layer containing peripheral blood mononuclear cells was clearly visible and collected using a pipette. Cells were washed twice with PBS and resuspended in complete RPMI media (RPMI, 10% FBS, 10% Pen/Strep, 2 mM/L-Glutamine) (Gibco, Life Technologies).

Immunofluorescence on primary cells

Cells were seeded in sterile glass coverslips in 24 well-plates and incubated overnight at 37ºC for adherence. After thalidomide/siRNA treatments and stimulation 25 mJ/cm2 (Bio- Link Crossliner BLX 312; Vilber Lourmat, Germany) in case of Keratinocytes or 10 ng/mL TNFα (Life Technologies) for PBMCs, cells were washed with PBS and then fixed for 15 minutes in 4% PFA followed by permeabilization with 0.1% TritonX-100 for 10 minutes. Blocking solution (BSA 5%) was added for 1 hour at RT and primary antibodies were incubated overnight at 4ºC and secondary antibodies were added for 2h at RT (Table S3). DAPI was used to visualize the nucleus. Images were captured using Olympus BX61 microscope.

RNA extraction

Skin biopsies were homogenized by politron and RNA was purified using miRVANA miRNA Isolation Kit (Applied Biosystems) following manufacturer's instructions. Total RNA from cultured cells was extracted after cell lysis with RNeasy Mini Kit (Qiagen). The yield and the quality of RNA from cell cultures were assessed by measuring its absorbance at 260nm and 280nm with Nanodrop. Ratios of A260/A280 between 1.8 and 2.1 were considered acceptable to use the RNA for the subsequent experiments.

RT-qPCR

Once RNA was obtained, 1ug of total RNA was reverse-transcribed into cDNA using the High Capacity cDNA Reverse Transcription Kit (Applied Biosystems) with the thermal cycler program: 25ºC for 10min, 37ºC for 120 min and 85ºC for 5min. Gene expression was assessed by TaqMan gene expression assays (FAM dye-labeled MGB probe, Applied Biosystems). Using 96 well plates or 384 well plates in the ABI PRISM 7000 or ABI PRISM 7900 thermocyclers respectively at 50ºC for 2 min, 95ºC for 10min, followed by 40 cycles of 95ºC for 15s and 60ºC for 1 min. Obtained data was normalized based on the expression of the endogenous control gene GAPDH (Hs02786624_g1).

Protein extraction and quantification

Protein was extracted from skin tissue following the instructions of PARIS kit (Thermo Fisher, Waltham, MA, USA). The skin was homogenized with the lysis buffer and samples were centrifuged at 12000 rpm at 4ºC for 3 minutes. The supernatant was collected, and 200 mL of chloroform was added. After 5 minutes on ice, samples were centrifuged 12000 rpm at 4ºC for 15 min. Then, the organic phase was collected, and isopropanol was added for 10 min at room temperature for protein precipitation. After centrifugation at 12000 rpm for 4ºC, 0.3 M of guanidine hydrochloride solution was added and centrifuged at 12000 rpm at 4ºC twice. Supernatant was discarded and the protein pellet was washed with ethanol and dried for 5-10 minutes. Finally, 1% SDS solution was added to dissolve the protein by repeated pipetting. The protein concentration was determined using the Bio-Rad Protein Assay (Bio-Rad, Hercules, CA, USA) according to the manufacturer's instructions.

Apoptosis and Proliferation Assays

Cells were plated in 24-well plates, stimulated with 25 mJ/cm2 in case of Keratinocytes or 10 ng/mL TNFα (Life Technologies) for PBMCs and thalidomide was added for 24h. Then, they were stained with Dead Cell Apoptosis Kit with Annexin V APC and SYTOX™ Green (Thermofisher) and measured by flow cytometry. For proliferation assays, cells were plated in 96-well plates, stimulated and thalidomide was added. After 24h, CyQUANT NF Cell Proliferation Assay Kit (Invitrogen) was used following manufacturer's instructions. Relative changes were calculated using "Non-Thalidomide treated cells" as control.

2. Supplementary Figures

Figure S1. PBMCs from patients were extracted pre and post-thalidomide (N=5). We obtained their RNA and we analyzed gene expression of CD8 Tcells (PRF1 and GRNZB) and iNKT (IL4 and INFG) related proteins by RT-qPCR. Relative expression were obtained using $2^{-\Delta\Delta Ct}$ method and GADPH as endogenous control. No significant differences were obtained but we observed a tendency in perforin A (PRF1, p=0.0674) and granzyme B (GRNZB, p=0.068).

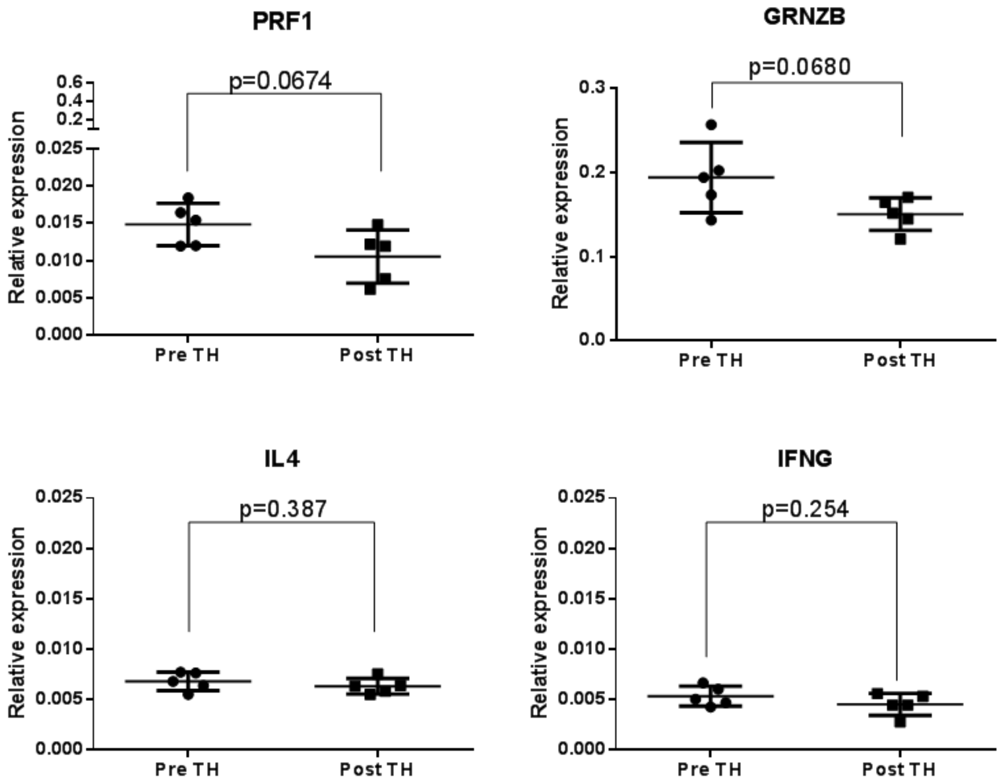

Figure S2. Topological analysis represents the relationships between proteins in the mechanism of action over cereblon modulation with the proteins identified as differential in RNA-seq analysis.

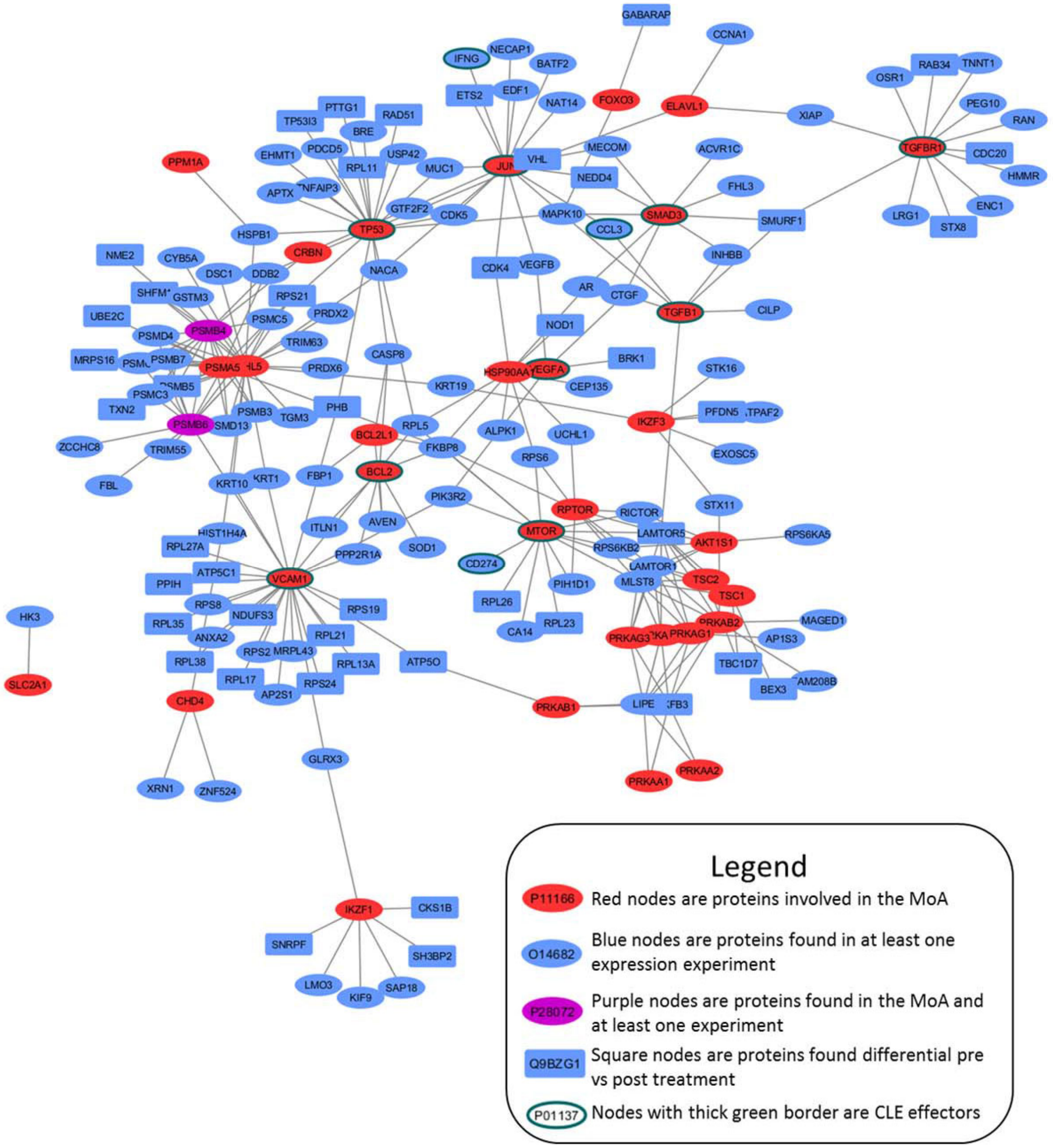

Figure S3. Topological analysis represents the relationships between proteins in the mechanism of action over IRF4 (cereblon-modulated protein) with the proteins identified as differential in RNA-seq analysis.

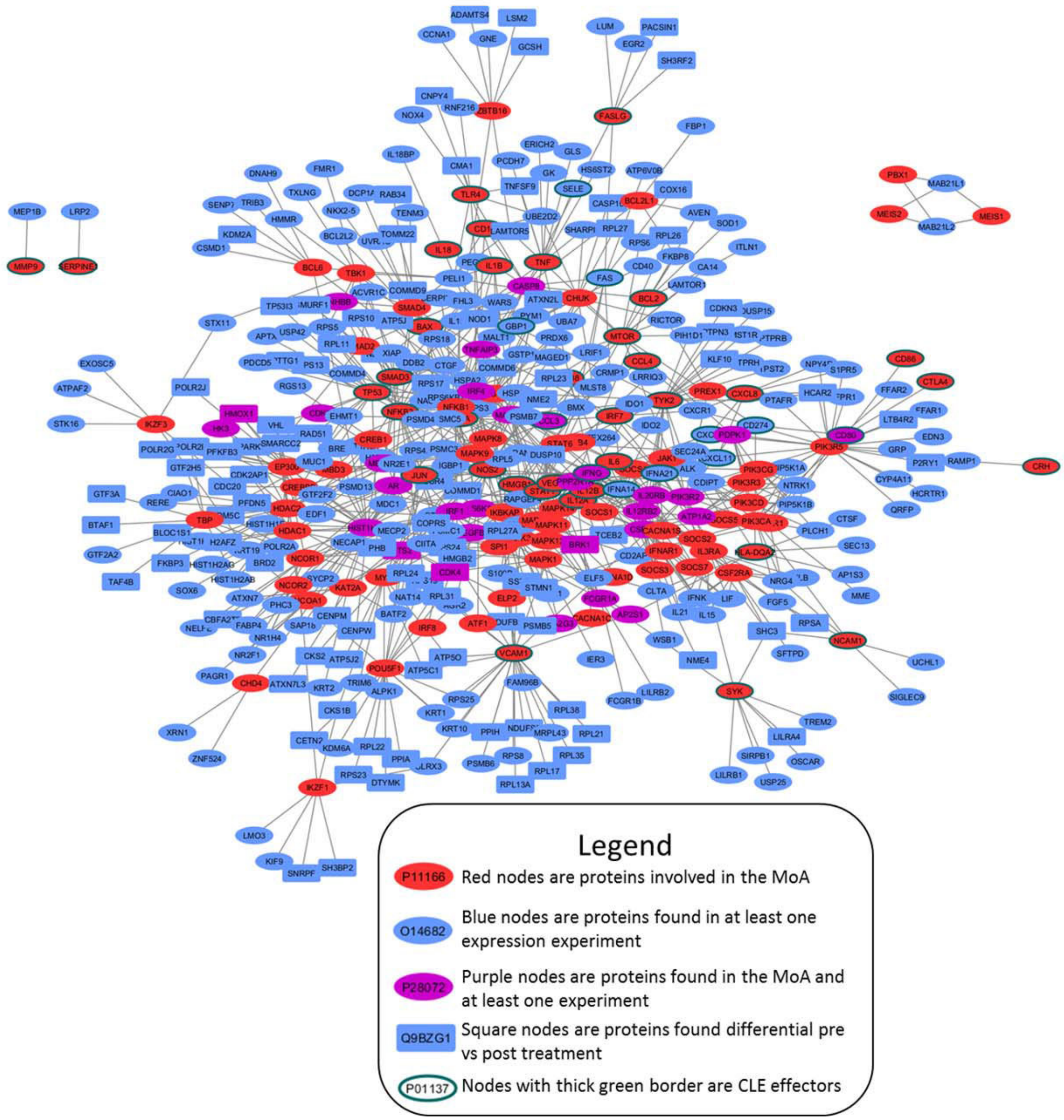

Figure S4. Western Blot of lysates from paired skin biopsies of Post-thalidomide and Pre-thalidomide treated patients for mTOR, NF-κB, CRBN and IRF4 (N= 3). β-actin was used as a control to normalize the levels of protein detected. Graphs show values in normalized band intensities between paired samples. * $p<0.05$ and ** $p<0.005$.

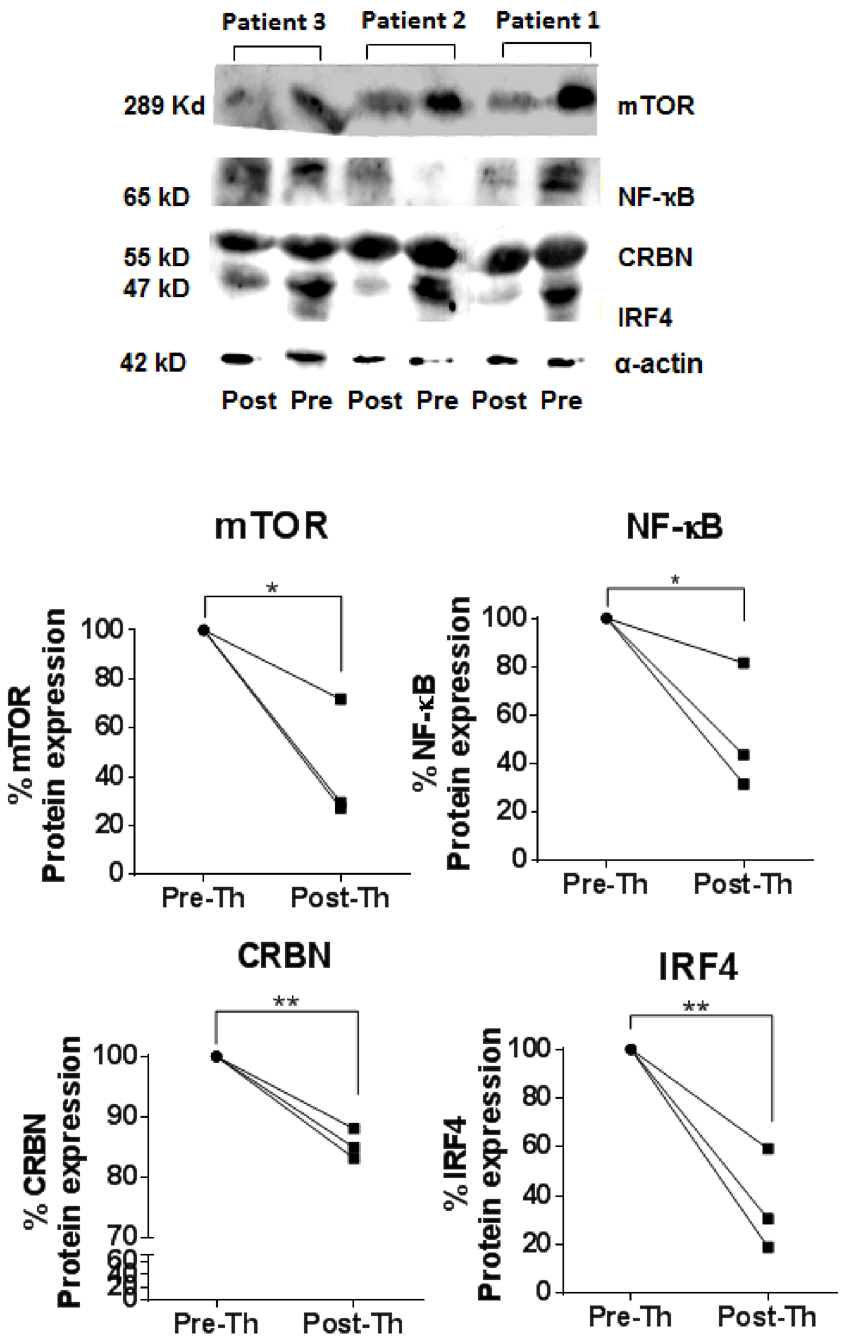

Figure S5. Immunofluorescence of mTOR in thalidomide-treated (Th) or non-treated (control) PBMCs. Nuclei was stained with DAPI (blue) and mTOR has green staining. Before treatment, PBMCs were stimulated with TNFα. No significant differences were observed between conditions (NS).

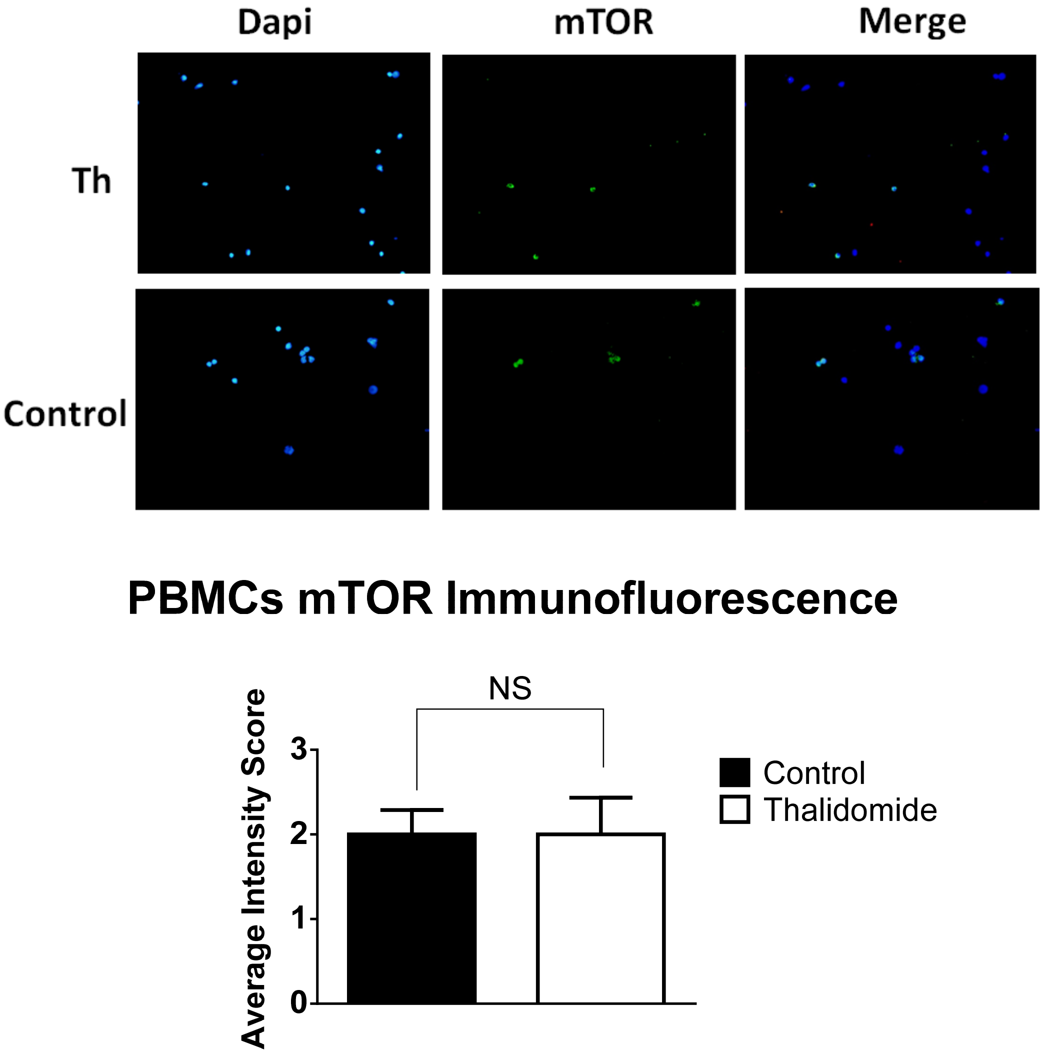

Figure S6. Proliferation in healthy PBMCs after thalidomide treatment. PBMCs were stimulated with TNFα overnight and treated with thalidomide (Th) or with sterile PBS, non-treated condition (Non-Th). After 6 hours, proliferation was quantified, and fold change was calculated over Non-Th condition. No significant differences were observed between conditions (NS).

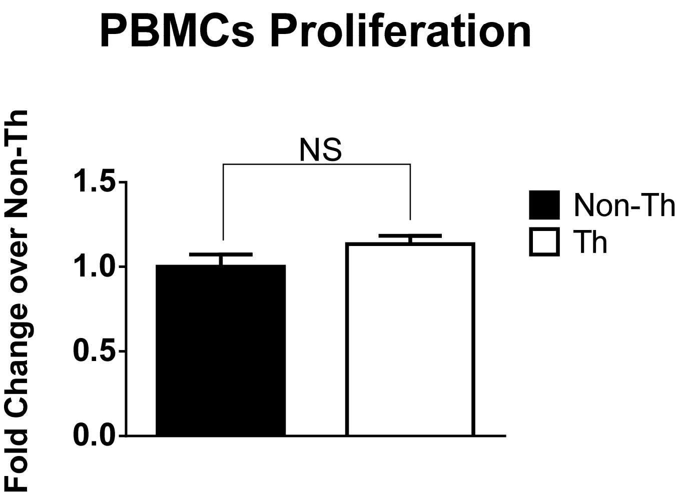

Figure S7. Immunofluorescence in thalidomide-treated (Th) or non-treated (control) PBMCs to monitoring autophagy. Nuclei was stained with DAPI (blue) and LC3 protein has green staining. Before treatment, PBMCs were stimulated with TNFα. No significant differences were observed between conditions (NS).

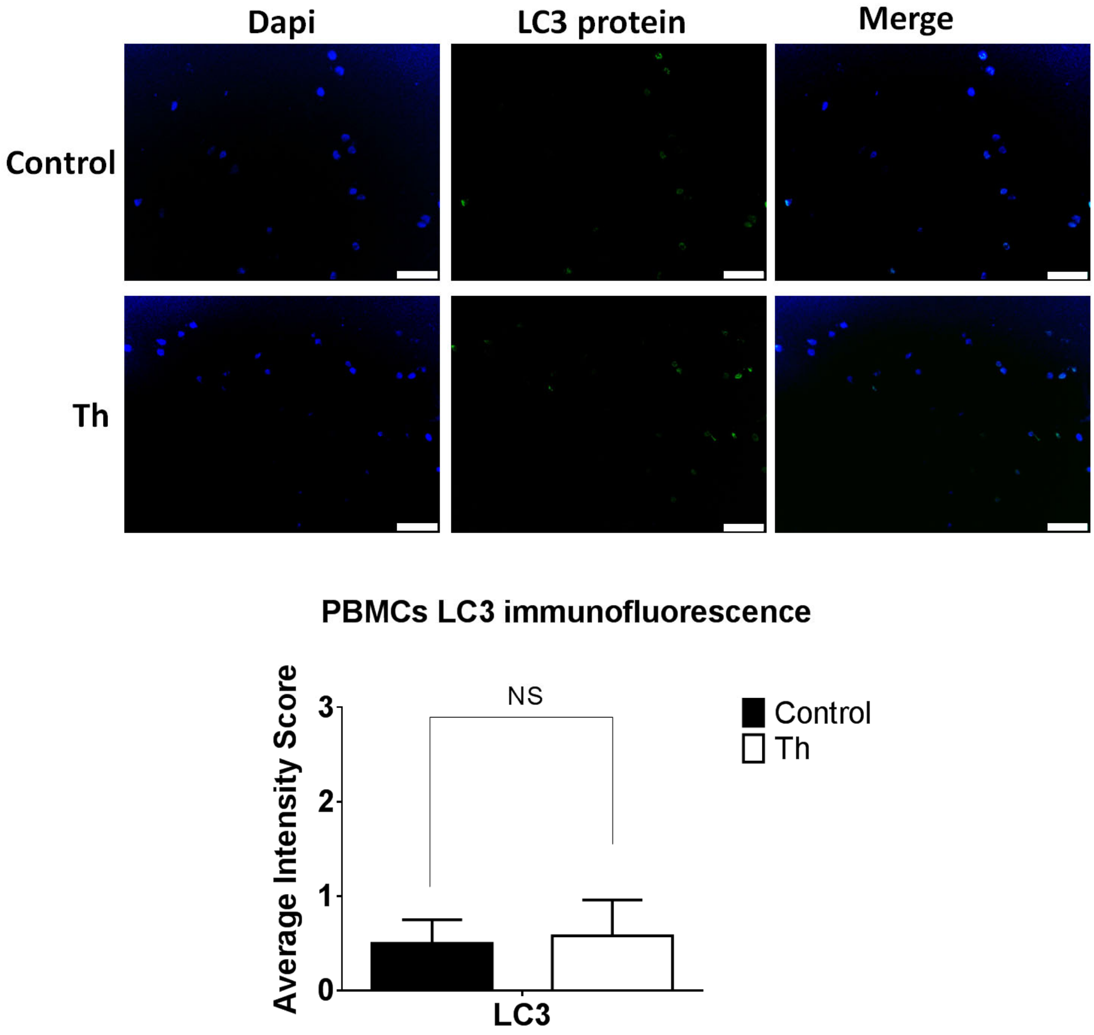

Figure S8. Western blot of cell based ubiquitination assay in keratinocytes treated or non-treated with thalidomide (Th). Results showed that AMPKa1 is ubiquinated in in absence of thalidomide and consequently AMPKa1 levels are decreased in comparison with thalidomide treated cells.

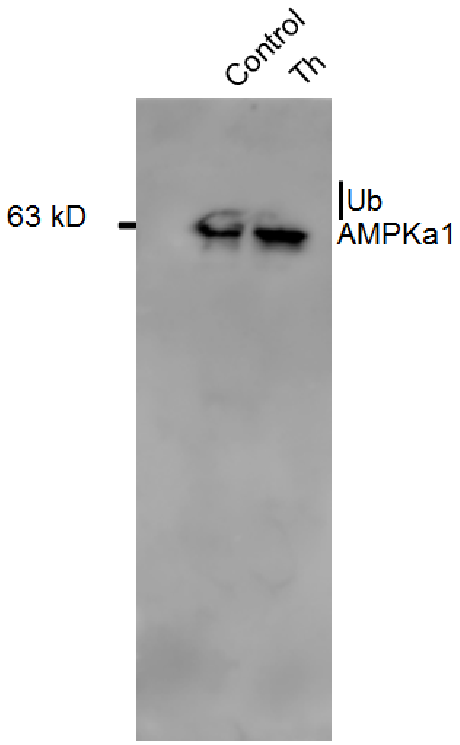

Figure S9. Proliferation and Apoptosis of Human epidermal keratinocytes after thalidomide addition. For proliferation and apoptosis measurement, cells were exposed to 25 mJ/cm^2 UVB and thalidomide was added for 24h (Th). For non-treated thalidomide cells (Non-Th), sterile PBS was added after 24 hours post-UVB stimulation. Fold change was calculated over Non-Th condition.

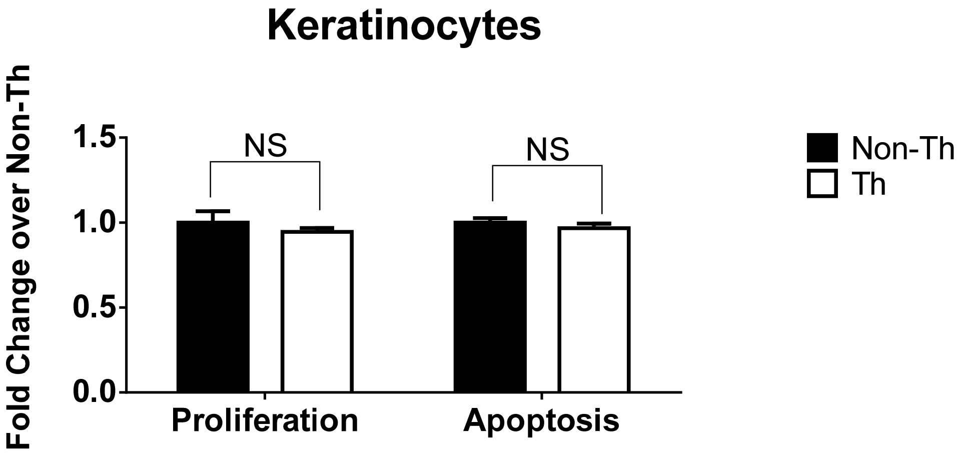

Figure S10. Immunofluorescence in thalidomide-treated (Th) or non-treated (control) keratinocytes to monitoring autophagy. Nuclei was stained with DAPI (blue) and LC3 protein has green staining. Before treatment, keratinocytes were stimulated with UV. No significant differences were observed between conditions (NS).

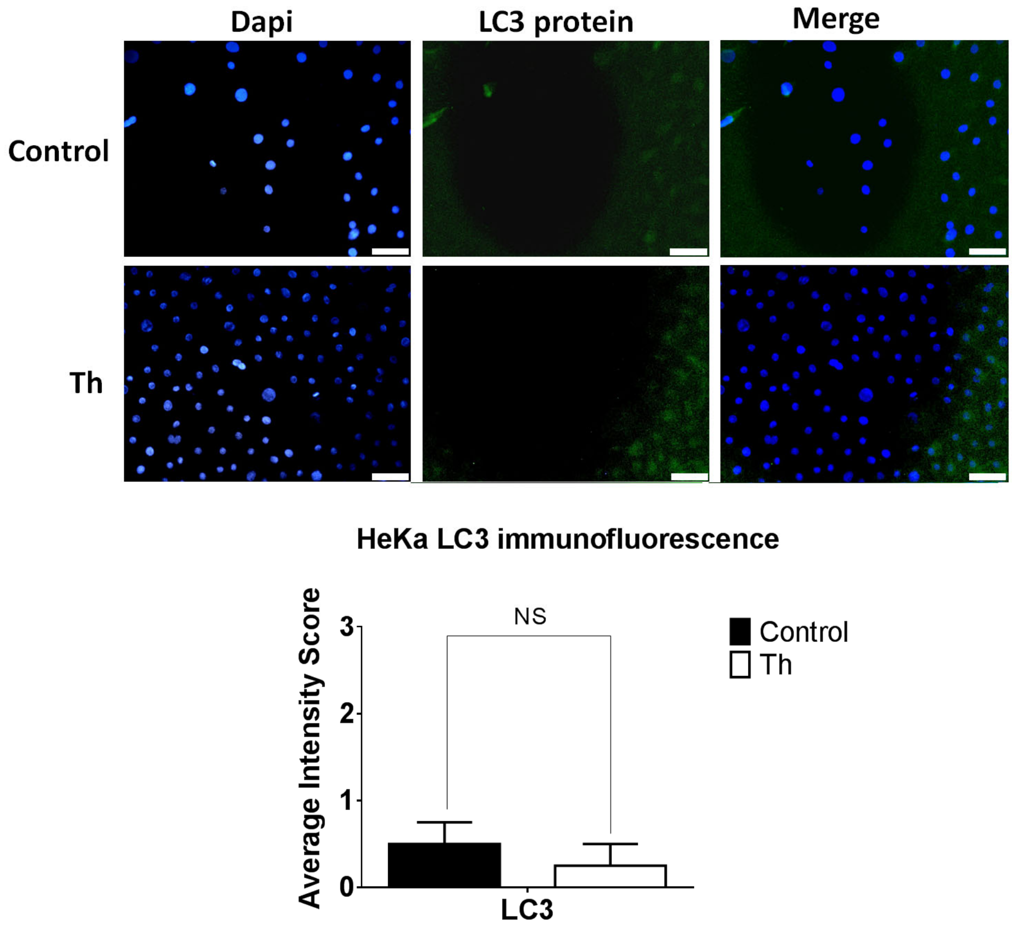

Figure S11. Immunofluorescence of NF-κB (green) in cultured keratinocytes with thalidomide or silenced IRF4. Counterstaining of nuclei is shown with DAPI in blue. No significant differences were observed between conditions (NS).

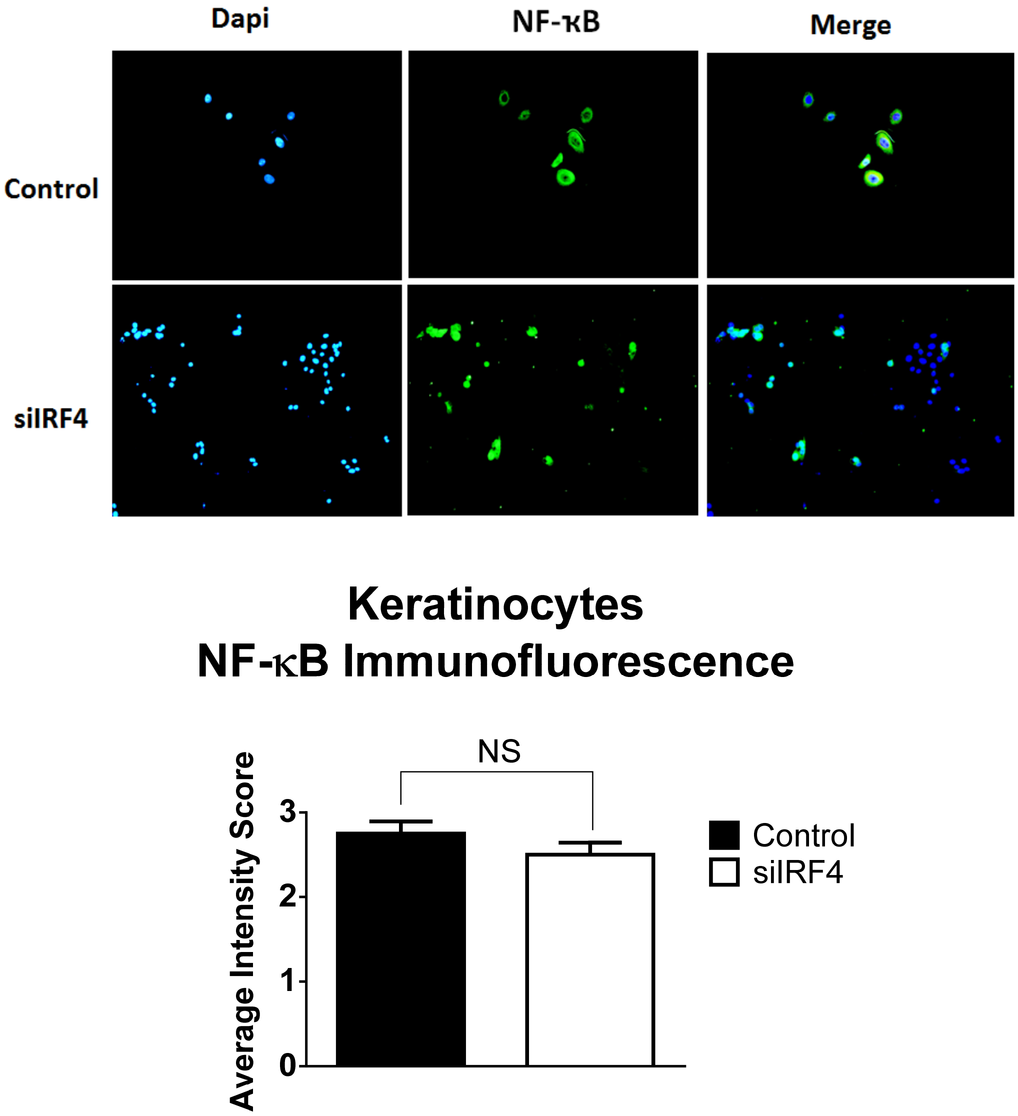

1. **Supplementary Tables**

Table S1. Clinical and laboratory characteristics of the study subjects.

	**CLE (n=10)**
AGE, mean (SD), yrs	44 (10.3)
Female, n (%)	10 (100%)
Photosensitivity, n (%)	3 (30%)
Smoking, (%)	4 (40%)
Type of CLE	
DLE	8 (80%)
SCLE	2 (20%)
CLASI ACTIVITY, mean (SD)	11.0±2.5
CLASI DAMAGE, mean (SD)	4.3±1.45
Systemic Lupus Erythematosus	4 (40%)
Clinical response to Thalidomide (4 weeks)<br>Complete response (CLASI=0)	7 (70%)
ANA antibodies positive, n (%)	8 (80%)
Anti-SSA/Ro antibodies positive, n (%)	1 (10%)

Values are number of patients and between brackets the percent of total number patients. The other values are means± SD. CLASI: Cutaneous Lupus Erythematosus Disease Area and Severity Index; CLE: cutaneous lupus erythematosus; DLE: discoid lupus erythematosus; SCLE: subacute cutaneous lupus erythematosus; ANA: Antinuclear Antibodies.

Table S2. Conjugated antibodies used in Flow Cytometry analysis.

**B cell subsets**	**Supplier**	**Code**
**CD19**	BD Biosciences	345788
**CD27**	BD Biosciences	558664
**CD38**	BD Biosciences	555460
**IgD**	BD Biosciences	555779
**T cell subsets**<br>**Thelper subsets**	**Supplier**	**Code**
**CD3**	BD Biosciences	340662
**CD4**	BD Biosciences	561842
**CD8**	BD Biosciences	555369
**CCR3**	BD Biosciences	561745
**CCR4**	BD Biosciences	744140
**CCR5**	BD Biosciences	560932
**CCR6**	BD Biosciences	564479
**CXCR3**	BD Biosciences	740183
**CD25**	BD Biosciences	340939
**FOXP3**	BD Biosciences	560046
**NK cell subsets**	**Supplier**	**Code**
**CD3**	BD Biosciences	340662
**CD16**	BD Biosciences	561842
**CD56**	BD Biosciences	555369
**6B11**	BD Biosciences	552825

Table S3. Antibodies used in Immunofluorescence, Immunohistochemistry and Western blot analysis.

**Primary Antibody**	**Supplier**	**Code**
**Anti-CRBN**	Abcam	ab244223
**Anti-IRF4 (MUM1)**	Abcam	ab133590
**Anti-MTOR**	Abcam	ab45989
**Anti-NF-KB p65**	Abcam	ab16502
**Anti-CD4**	Roche	SP35
**Anti-CD8**	Agilent	DK25
**Anti-CD56**	Fisher Scientific	56C04
**Anti-6B11**	Invitrogen	14-5806-82
**Anti-Phospho-Raptor (Ser863)**	Invitrogen	PA5-64849
**Anti-AMPK alpha-1**	Invitrogen	AHO1332
**Anti-Ubiquitin**	Abcam	Ab7780
**Secondary Antibody**	**Supplier**	**Code**
**Alexa-488-conjugated anti-rabbit IgG**	Abcam	ab150077
**Alexa-647-conjugated anti-mouse IgG**	Abcam	ab150115

Table S4. Primer IDs used in Taqman RT-qPCR from Applied Biosystems.

Gene	Assay ID (TaqMan)
**GADPH**	Hs02786624_g1
**NFKB1**	Hs00765730_m1
**MTOR**	Hs00234508_m1
**CXCL1**	Hs00236937_m1
**IL1B**	Hs01555410_m1
**CCL3**	Hs00234142_m1
**GATA3**	Hs00231122_m1
**TBX21 (T-bet)**	Hs00894392_m1
**TGFB1**	Hs00998133_m1
**IL-2**	Hs00174114_m1
**CXCL8 (IL-8)**	Hs00174103_m1
**TNF**	Hs00174128_m1
**PRKAA1 (AMPKa1)**	Hs01562308_m1
**IL-10**	Hs00961622_m1
**IFNA1**	Hs04189288_g1
**IL4**	Hs00174122_m1
**IFNG**	Hs00989291_m1
**PRF1**	Hs00169473_m1
**GRNZB**	Hs00188051_m1

Table S5. Characterization of Cutaneous Lupus Erythematosus (CLE) includes 206 proteins distributed in the named motives.

MOTIVE NAME	Number of proteins
Innate immune system activation	75
Keratinocyte apoptosis	23
Impaired apoptotic clearance	10
Autoantigen exposure	16
Lymphocyte recruitment and activation	44
Complement response and IgG complex formation	15
Fibrosis – Discoid Lupus Erythematosus	22
Angiogenesis	1

Table S6. Detail of the targets identified for thalidomide and the use of the targets in the different project steps.

Protein name	Gene name	UniProt code	Reference	Use
**Protein cereblon**	CRBL	Q96SW2	PMID: 27460676	Model training and MoA target
**DNA-binding protein Ikaros**	IKZF1	Q13422	PMID: 27492707	Model training and MoA target
**Zinc finger protein Aiolos**	IKZF3	Q9UKT9	PMID: 27492707	Model training and MoA target
**Interferon regulatory factor 4**	IRF4	Q15306	PMID: 26269456	Model training and MoA target
**Homeobox protein Meis2**	MEIS2	Q14770	PMID: 27492707	Model training and MoA target
**Alpha-1-acid glycoprotein 1**	ORM1	P02763	PMID: 8755512	Model training
**Alpha-1-acid glycoprotein 2**	ORM2	P19652	PMID: 8755512	Model training
**Fibroblast growth factor 2 (FGF-2)**	FGF2	P09038	PMID: 26503997; PMID: 25053990	Model training

Table S7. MoA included 27 proteins with 14 proteins related directly with CLE (in bold).

PROTEIN	Relationship with CLE
**VEGFA**	Higher VEGF levels in CLE
**VCAM1**	High VCAM-1 expression on endothelium tissue in CLE lesions
**MTOR**	MTOR is involved in UVB signaling in keratinocytes and is a potential target for CLE
**TLR4**	TLR4 increases inflammatory response and has been associated to autoimmunity in CLE
**TNF**	TNF-α may have both inflammatory and immunomodulatory roles in CLE
**IL6**	Interleukins are induced by UVB in keratinocytes in CLE
**CXCL8**	Interleukins are induced by UVB in keratinocytes in CLE
**TGFB**	High levels in DLE lesions
**JUN**	In CLE, JUN has been associate to UVB exposure
**CD14**	CD14+ macrophages are increased in DLE skin
**CCL3**	CCL3 are found upregulated in CLE
**MMP9**	MMP9 has been linked to a time-dependent TGF-β-related scarring process in DLE
**NFKB1**	In CLE, NFKB1 has been associated to UVB exposure, and to UVB-induced TNF-α expression
**IRF4**	DC dysfunction in lupus-prone mice relies on IRF4 pathways
**AMPK**	Not related
**IKZF1**	
**IKZF3**	
**CHD4**	
**ELAVL1**	
**RPTOR**	
**TSC1**	
**TSC2**	
**AKT1S1**	
**CRBN**	
**SPI1**	
**BCL6**	
**RELA**	
